# Integrative Informatics Analysis of Transcriptome and Identification of Interacted Genes in the Glomeruli and Tubules in CKD

**DOI:** 10.3389/fmed.2020.615306

**Published:** 2021-02-12

**Authors:** Lingyun Liu, Fuzhe Ma, Yuanyuan Hao, Zhengzi Yi, Xiaoxia Yu, Bo Xu, Chengguo Wei, Jinghai Hu

**Affiliations:** ^1^Department of Andrology, The First Hospital of Jilin University, Jilin, China; ^2^Department of Nephrology, The First Hospital of Jilin University, Jilin, China; ^3^Department of Urology, The First Hospital of Jilin University, Jilin, China; ^4^Division of Nephrology, Icahn School of Medicine at Mount Sinai, New York, NY, United States; ^5^Division of Nephrology, Affiliated Zhongshan Hospital of Dalian University, Dalian, China

**Keywords:** informatics analysis, CDK, DKD, glomeruli tubule, crosstalk

## Abstract

Chronic kidney disease (CKD) is a complex disease in which the renal function is compromised chronically. Many studies have indicated the crosstalk between the tubule and the glomerulus in CKD progression. However, our understanding of the interaction of tubular and glomerular injury remains incomplete. In this study, we applied a meta-analysis approach on the transcriptome of the tubules and glomeruli of CKD patients to identify differentially expressed genes (DEGs) signature. Functional analysis of pathways and Gene Ontology found that tubular DEGs were mainly involved in cell assembly and remodeling, glomerular DEGs in cell proliferation and apoptosis, and overlapping DEGs mainly in immune response. Correlation analysis was performed to identify the associated DEGs in the tubules and glomeruli. Secreted protein comparison and verification experiments indicated that WFDC2 from the tubule could downregulate PEX19 mRNA and protein levels at the glomeruli in diabetic kidney disease (DKD). This study revealed the distinctive pathways of the tubules and glomeruli and identified interacted genes during CKD progression.

## Introduction

Chronic kidney disease (CKD) affects between 8 and 16% of the population worldwide and is often underrecognized by patients and clinicians (1, 2). Diabetic kidney disease (DKD) is the leading cause of CKD and is the single strongest predictor of mortality in patients with diabetes (3). In recent years, although the development of clinical therapy for DKD has made great progress, the progression of DKD still cannot be controlled (4). Therefore, more detailed study of CKD-associated mechanisms is needed to fully understand its clinical relevance and underlying pathophysiology, which is critical to identify predictors of the disease course and therapeutic targets.

Traditional studies have identified multiple individual factors involved in the pathogenesis of CKD (5–7). However, these candidate gene approaches have limited value toward the full understanding of the molecular mechanisms of these diseases. Recent studies have provided us new insights into the crosstalk between tubular and glomerular segments (8). Glomerulosclerosis with resulting ischemia to the downstream tubules causes tubulointerstitial fibrosis (9). Tubulointerstitial injury may also lead to increased glomerular injury (10). A sequential tubular–glomerular injury model found that even mild preexisting tubulointerstitial injury sensitized the glomeruli to subsequent podocyte-specific injury (11). Many studies also indicated that tubular epithelial cells (TECs) and glomerular endothelial cells (GECs) can crosstalk with each other in the development of DKD. Studies have shown that TECs inflammatory response (12), Ang-1/Ang-2 Tie2 (13), and VEGF/VEGFR axis (14) contribute to the injury of GECs, whereas Kruppel-like factor (KLF) (15), HGF/c-MET (16), and IGFBPs (17) mediate injury from GECs to TECs. Improving injury and maintaining normal crosstalk between them may become a new strategy for the prevention and treatment of kidney diseases in the future.

In this study, we applied a meta-analysis and correlation analysis to identify genes and pathway signatures for the tubule and glomerulus and novel genes crosstalk between them. We used a meta-approach on CKD patients vs. healthy donors to identify differentially expressed genes (DEGs) signature. Functional analysis of pathways and Gene Ontology (GO) was performed to identify overlapping and distinguish pathways for the tubule and glomerulus in CKD. Correlation analysis was also performed with gene expression in both the tubule and glomerulus tissues to obtain the interaction genes. Secreted proteins were compared with the interaction gene pairs, and we identified that WFDC2 and PEX19 could be interacted from the tubules and glomeruli within the pathophysiological progression of CKD.

## Methods

### Data Collection

Publicly available human microarray and next-generation sequencing datasets for all kidney diseases [lupus nephritis (LN), diabetic nephropathy (DN), focal segmental glomerulosclerosis (FSGS), membranous nephropathy (MN), IgA nephropathy (IgAN), and minimal change disease (MCD)] were obtained from Nephroseq (https://www.nephroseq.org/) and PubMed and downloaded from GEO (Supplementary Table 1). All the transcriptome data were downloaded from GEO, and the accession numbers were listed in Supplementary Table 1. We collected eight datasets for kidney diseases, which included high-throughput transcriptome data for 508 disease and control samples. Each dataset manually selected the samples with clinical information. There are two datasets that contained the transcriptome data for tubule tissue, two datasets for glomerular transcriptome, and four datasets for both tubule and glomerulus data for the same patient (Supplementary Table 1). For each study, we grouped the samples with the clinical and phenotypic information reported by the corresponding original studies. Then, for the raw microarray data, we performed quality assessment, and all the microarray platform data were re-annotated to the most recent NCBI Entrez Gene Identifiers (Gene IDs) by AILUN (http://ailun.ucsf.edu) (18). All the expression values were base-two log-transformed and normalized by quantile–quantile normalization.

### Meta-Analysis

Meta-analysis methods were described in our previous paper (19). Briefly, we used two meta-analysis methods effect sizes and combining significance analysis of microarrays (SAM) *q* values to analyze all the transcriptome data. In the first method, we estimated the effect size and summarized the effect size with fixed effect inverse–variance model for all annotated genes in all datasets. We combined the study-specific effect sizes for each gene into one meta-effect size (f_meta_) using a linear combination of effect sizes (fi) by weighting each effect size by the inverse of the variance (wi) in the corresponding study (19). We used false discovery rate (FDR) (20) to test the significant difference for each gene as FDR ≤5% was used for cutoff as significant. In the combining SAM (21) method, we used *q* <10% as cutoff for significantly expressed genes between healthy controls and CKD patients. Finally, for different datasets, we used Fisher's exact test to test whether the probability of obtaining was significant or not with *p* ≤ 0.05 as cutoff.

### Pathway Network, Generation, and Analyses

The DEGs for microarray and sequencing in kidney diseases compared with normal were identified by meta-analysis. Then, DEGs for the tubules and glomeruli were compared to obtain the unique and overlapping DEGs. We used two methods to perform gene enrichment analysis. DEGs with a fold change cutoff of ≥1.5 were used INGENUITY IPA (www.ingenuity.com/products/ipa) and Enrichr (https://amp.pharm.mssm.edu/Enrichr/) for GO and pathways. The interaction of genes was visualized by Cytoscape (https://cytoscape.org/).

### Crosstalk Between Tubular Cell and Podocyte in Disease Condition

Next, for the four datasets having both tubular and glomerular data from the same patient, we performed the gene expression correlation analysis with “pearson,” “kendall,” and “spearman” correlation coefficient methods. We identified specific correlated paired genes with correlation coefficient >0.7 and *p* < 0.001 as cutoff. Then, we compared the correlated DEG pairs and obtained 59 pairs of associated DEGs in all four datasets. The association between the tubules and the glomeruli was visualized with Cytoscape (http://www.cytoscape.org/). Then, we obtained the human secretome and membrane proteome list from Human Protein Atlas (www.proteinatlas.org) (22) and identified the secreted proteins in our associated gene pairs, which could be secreted and interacted with proteins in other cells.

### Cell Culture, Real-Time PCR, and Western Blot

HK2 cell (ATCC CRL-2190) and glomerular epithelial cell (ATCC CRL-192) obtained from ATCC were cultured in RPMI-1640 medium (Corning). Human podocyte cell line (23) was cultured in RPMI-1640 medium (Corning) containing 10% fetal bovine serum (FBS; Corning) supplemented with 1% Insulin–Transferin–Selenium-A liquid media (Life Technologies) and 100 U/ml penicillin. Cultures were incubated at a 33°C humidified incubator and transferred at 37°C for differentiation (23). Expression-ready lentiviral constructs for WFDC2 overexpression were purchased from Horizon Inspired Cell Solutions (MHS6278-202801004, Clone Id: 5186932, MGC Human WFDC2 Sequence-Verified cDNA). Negative control overexpression pCMV-SPORT6 was used as a negative experimental control. Lentivirus for WFDC2 and control plasmids has been produced by HEK 293T cells. TRIzol reagent (Thermo Fisher Scientific) was used to extract RNA following the manufacturer's protocol for cultured cell and mice kidneys. Quantitative real-time PCR and 2^−ΔΔ*CT*^ method were performed to quantity the gene expression. For western blot, cultured cells and mice kidney tissues were lysed with lysis buffer with phosphorylation protease and protease inhibitor cocktails. The following antibodies were used: WFDC2 (rabbit monoclonal HE4/WFDC2, Catalog # NBP2-66883; Novus Biologicals), PEX19 (PEX19 monoclonal antibody (GT554), Catalog # MA5-17266; Invitrogen), and GAPDH (mouse monoclonal antibody, Catalog # G8795-100UL; Sigma).

### STZ-Induced Diabetic Mice Model and Glomeruli Isolation

eNOS^−/−^ mice were purchased from Jackson Laboratory, and streptozotocin (STZ)-induced model and glomeruli isolation were described previously (24). For induction of diabetes, 8 weeks old male eNOS^−/−^ mice were injected low-dose STZ (Sigma-Aldrich) for 5 consecutive days at 50 μg/g intraperitoneally. The same age male CL-eNOS^−/−^ mice injected with vehicle were used as non-diabetic controls. The diabetes group model was considered successful when the fasting blood glucose level was higher than 300 mg/dl after 10 weeks of STZ injection. The glomeruli and tubules were separated using Dynabead perfusion as described in a previous paper (25). Briefly, mice were perfused with phosphate-buffered saline (PBS) for 2 min then with prewarmed 8 ml bead solution in enzymatic digestion buffer (Collagenase type II 300 U/ml, Proteinase E 1 mg/ml, and DNase I 50 U/ml). The kidneys were chopped to 1 mm^3^ pieces and digested at 37°C for 15 min in a digestion buffer with continued rotation. A 100-μm cell strainer was used to get rid of the undigested tissue debris then centrifuged at 200 g to obtain the tubules and glomeruli. The cell pellet was resuspended in Hanks' balanced salt solution, and the glomeruli were collected using a magnet. The rest of the non-glomeruli part was collected for the tubule part. The separated glomeruli and tubules were resuspended in Hanks' buffer for further cell lysis for quantitative PCR (qPCR) and western blot.

## Results

### Meta-Analysis of Transcriptome Reveals Different Molecular Mechanisms for Tubular and Glomerular Tissues of CKD

**Figure 1:**
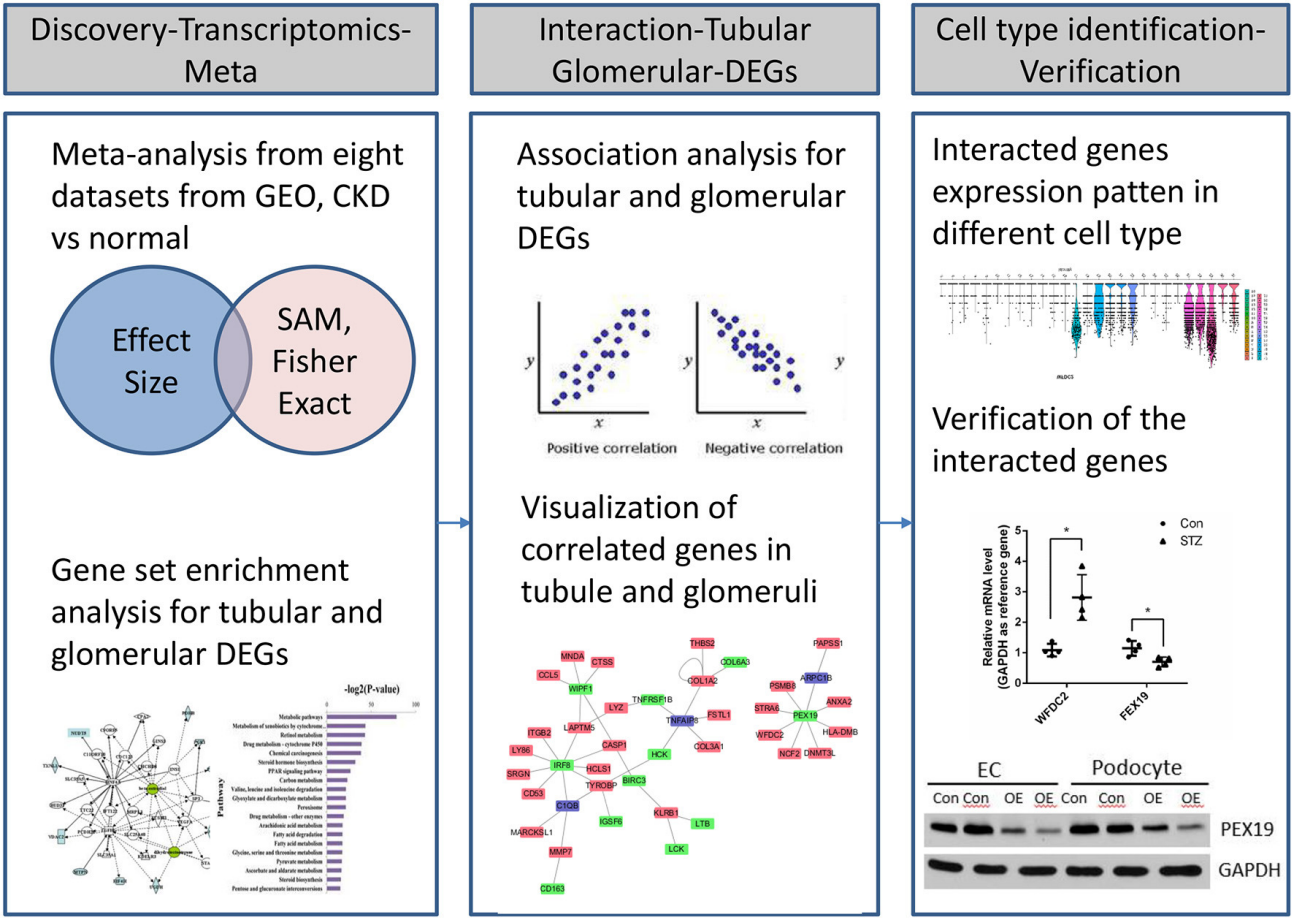
Workflow of transcriptional profiling and identification of interacted genes through integrative informatics approach.

We obtained transcriptome data from eight studies with both tubular and glomerular samples of renal CKD patients (Supplementary Table 1). We used the clinical information reported from the corresponding studies. Finally, transcriptome data of 508 samples of healthy control and CKD patients were used for further analysis. Preprocessing analysis was performed for gene annotation, quantile–quantile normalization (18). We applied two meta-analysis methods (see the Methods section, Figure 1) to obtain the DEGs in CKD vs. normal samples in tubular and glomerular tissues across multiple datasets. Based on two meta-analysis methods, we identified a total of 619 (for the glomeruli, Supplementary Table 2) and 1,824 (for the tubules, Supplementary Table 3) overlapped genes to be significantly different. Meta-effect size, meta-SAM *q* values, effect size for each dataset, and SAM *q* values for each dataset were shown in Supplementary Tables 2, 3. Of these DEGs, there are 196 overlapping genes for tubular and glomerular samples, and 1,628 and 423 DEGs unique for tubular and glomerular samples (Supplementary Table 4).

**Figure 2:**
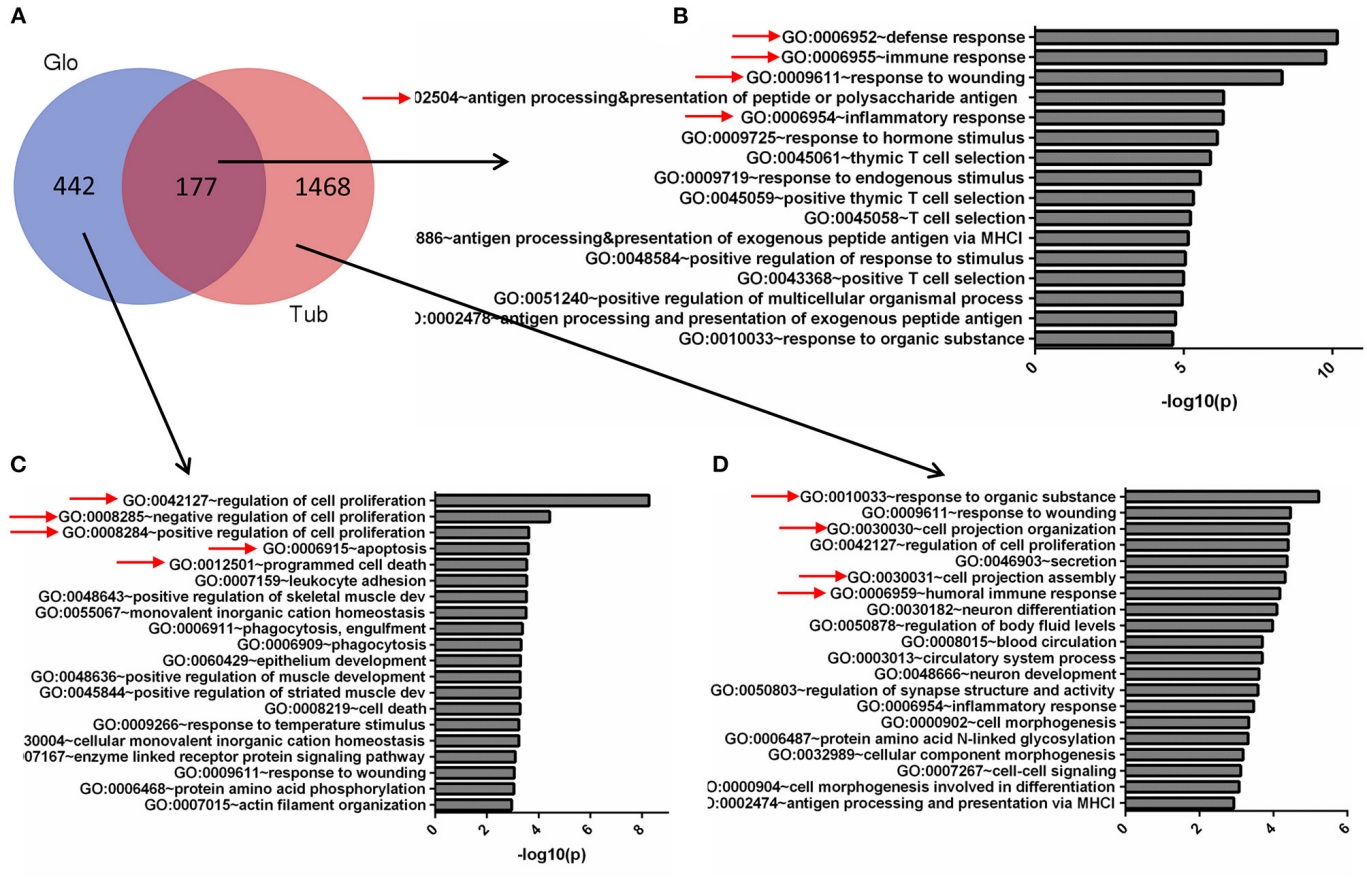
Gene Ontology (GO) terms of glomerular and tubular DEGs. **(A)** Venn diagram for the overlapping comparison of glomerular and tubular DEGs. GO terms of **(B)** glomerular and tubular overlapping DEGs, **(C)** tubular unique DEGs, and **(D)** glomerular unique DEGs. Significance is expressed as a *p*-value calculated using Fisher's exact test (*p* < 0.05) and shown as –log_10_ (*p*-value).

To compare the DEGs functions for the tubules and glomeruli in CKD, we performed pathway and network analysis for the tubular and glomerular common and unique genes using QIAGEN's Ingenuity Pathway Analysis. We found that tubular and glomerular DEGs were involved in distinct pathways and GO terms (Figure 2A). The common DEGs in tubular and glomerular samples were mainly involved in immune response (Figure 2B), with GO terms of defense response, immune response, and response to wounding, highlighted in Figure 2B (Supplementary Table 5). The glomerular DEGs regulated cell proliferation and apoptosis (Figure 2C, Supplementary Table 7) with many GO terms of regulation of cell proliferation and cell death, whereas the tubular DEGs were mainly involved in cell assembly and secretion (Figure 2D, Supplementary Table 6). The genes include FCN1, C1QB, ITGAM, and WIPF1, which are well-known to be involved in kidney injury (26–29).

**Figure 3:**
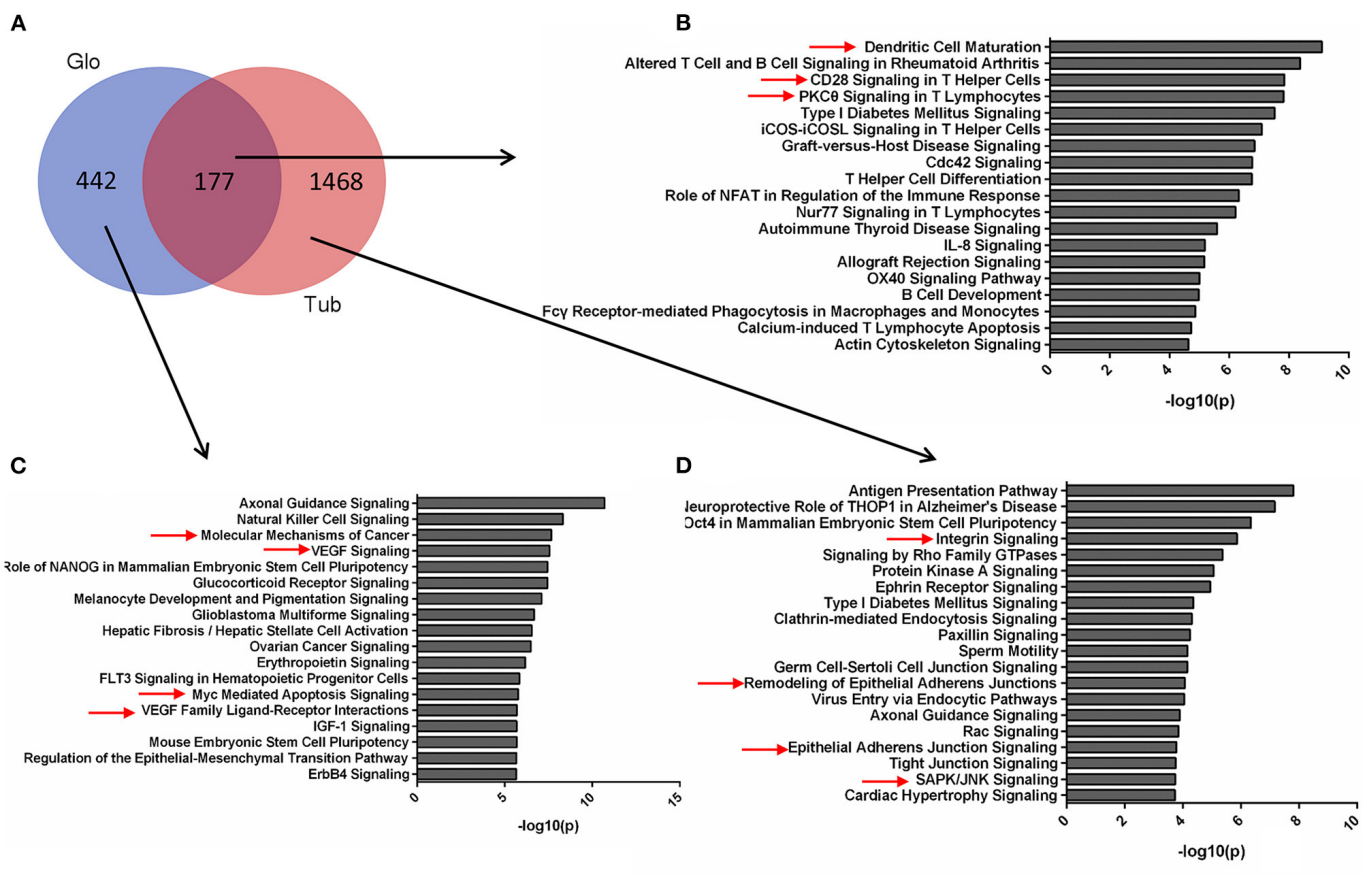
Pathways for tubular and glomerular DEGs. **(A)** Venn diagram for the overlapping comparison of glomerular and tubular DEGs. Pathways of **(B)** glomerular and tubular overlapping DEGs, **(C)** tubular unique DEGs, and **(D)** glomerular unique DEGs. Significance is expressed as a *p*-value calculated using Fisher's exact test (*p* < 0.05) and shown as –log_10_ (*p*-value).

The pathways for tubular and glomerular DEGs were also distinguished (Figure 3A). The pathways common for tubular and glomerular DEGs were involved in immune response, with dendritic cell maturation, altered T cell and B cell signaling in rheumatoid arthritis, and CD28 signaling in T helper cells (Figure 3B, Supplementary Table 8). The pathways for glomerular DEGs were involved in VEGF signaling, molecular mechanisms of cancer, and Myc-mediated apoptosis signaling (Figure 3C, Supplementary Table 9). The tubular DEGs were enriched in different pathways, such as integrin signaling, remodeling of epithelial adherents junctions, and SAPK/JNK signaling (Figure 3D, Supplementary Table 10). The pathways for tubular and glomerular DEGs are consistent with GO terms. The common DEGs pathways are involved in immune response, and the pathways for tubular DEGs are mainly involved in cell remodeling and assembly. The pathways for the glomeruli are involved in cell proliferation and apoptosis as pathways related to cancer and apoptosis signaling.

### Gene Expression Correlation Analysis Between the Tubule and the Glomerulus

**Figure 4:**
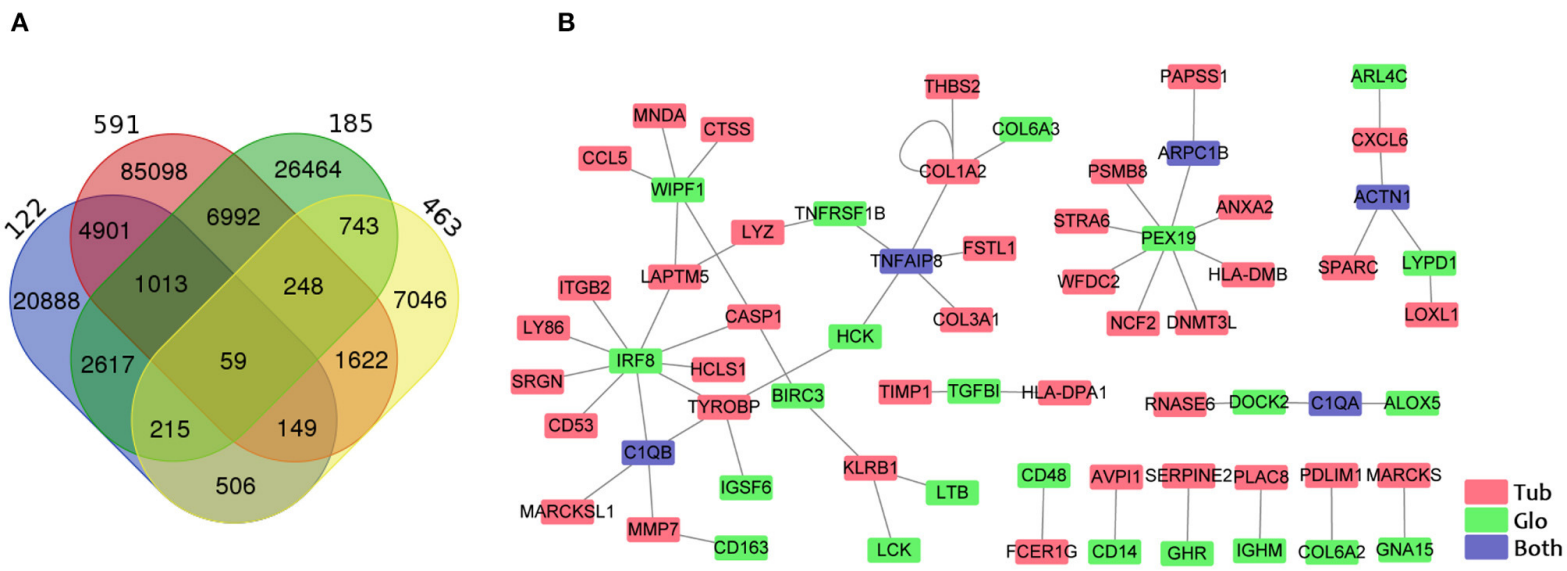
Identification of genes correlated between the tubule and the glomerulus. **(A)** Venn diagram shows the overlapping of associated gene pairs in four datasets. **(B)** Visualization of 59 paired genes correlated in the tubules and glomeruli in all four datasets. Red, green, and blue colors indicate the GECs in the tubules, glomeruli, and both the tubules and the glomeruli.

Some previous studies have delineated that glomerular injury causes tubulointerstitial injury, and that tubular injury sensitizes the glomeruli to injury (9, 11, 30). To identify the crosstalk between tubular and glomerular DEGs in CKD patients, we performed the gene co-expression correlation analysis for the four datasets with tubular and glomerular expression data in the same patient. We identified 59 specific correlated paired genes with a cutoff of correlation coefficient >0.7 and *p* < 0.001 in all four datasets (Figure 4A, Supplementary Table 11). Visualization of the networks from these correlated DEG pairs was generated by Cytoscape (Figure 4B). We found that some interesting associated genes from the tubules and glomeruli reported could be interacted by previous studies. IRF8 from the glomerulus positively correlated with many genes in the tubule, such as C1QB, CASP1, CD53, HCLS1, ITGB2, LAPTM5, LY86, SRGN, and TYROBP. Studies have proven that IRF8, a pro-apoptotic factor, was a hypomethylated gene in acute kidney injury (AKI) and this hypomethylation was associated with a marked induction of Irf8 (31). Studies showed that IRF8 is the transcription factor that regulates C1QB (32), CASP1 (33), CD53 (34), LAPTM5 (35), and TYROBP (36) as binding to their promoter regions.

### Identification and Verification of Secreted Proteins From Tubular and Glomerular Interaction

**Figure 5:**
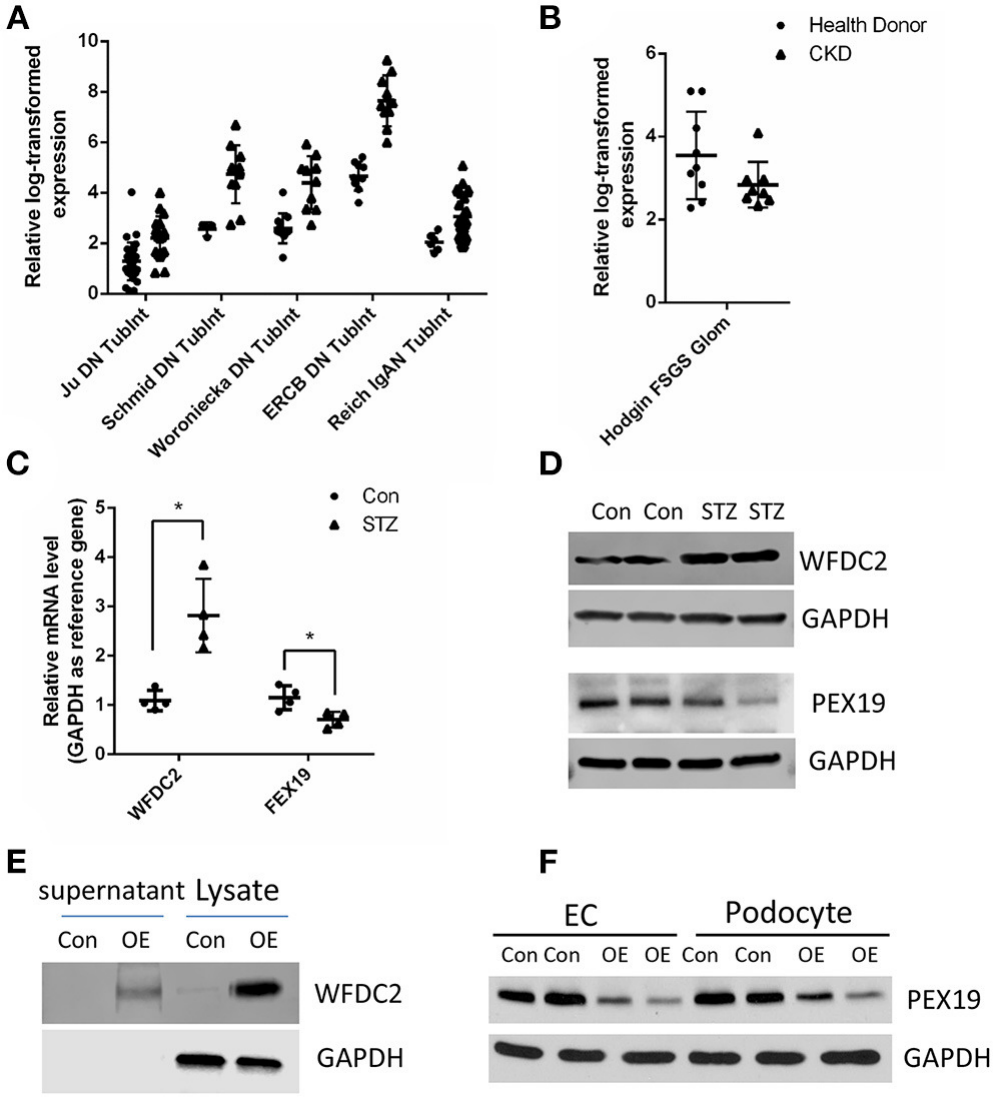
Verification of WFDC2 and PEX19 interaction in HK2, podocyte, and glomerular endothelial cells. **(A)** Relative WFDC2 transcript levels were upregulated in many diabetic nephropathy human and mice datasets in the kidney tubule. **(B)** Relative FEX19 transcript levels were downregulated in FSGS in the kidney glomeruli in Nephroseq database. **(C,D)** WFDC2 in the tubule was upregulated, and FEX19 in the glomeruli was downregulated in STZ-induced diabetic mice by qPCR and western blot. **(E)** Western blot shows WFDC2 overexpression in HK2 cell. **(F)** PEX19 protein level was decreased with WFDC2 treatment in podocyte and glomerular endothelial cells by western blot. Con, WFDC2 empty vector; OE, WFDC2 overexpression vector; EC, glomerular endothelial cells. **(A–C)** Values are mean ± SEM; *p ≤ 0.05 between means. **(A,B)** All the genes were significantly changed in CKD compared with control with *p* < 0.001.

Next, we tried to identify the secreted proteins from our tubular and glomerular associated gene pairs as the secreted proteins can interact with proteins in other cells. From public data at Human Protein Atlas portal (www.proteinatlas.org), we obtained 1,708 predicted secreted proteins. Overlapping with our gene pairs, we identified 18 and 10 secreted proteins in tubular and glomerular samples in CKD (Supplementary Table 12). Many proteins are well-known to be important in kidney diseases, such as TGFBI (37), TNFRSF1B (also known as TNFR2) (38, 39), CXCL6 (40), and CCL5 (41). In the secreted proteins list, we found that WFDC2 from the tubule was negatively correlated with PEX19 from the glomeruli (Supplementary Table 12). WFDC2 is a molecular marker of tubulointerstitial fibrosis and tubular cell damage in patients with CKD (42–44). We then validated that WFDC2 was significantly upregulated in many kidney diseases in human and kidney disease models in mouse from Nephroeseq database (Figure 5A). FEX19 was downregulated in FSGS in the glomeruli in Nephroeseq (Figure 5B). We also validated that WFDC2 is mainly expressed in tubular cells in single-cell sequencing data from Nephrocell database (http://nephrocell.miktmc.org) (Supplementary Figure 1). Next, we found that WFDC2 was upregulated, whereas FEX19 was downregulated in STZ-induced eNOS depletion diabetic mice model by qPCR (Figure 5C) and western blot (Figure 5D). To further examine the regulation of WFDC2 to FEX19, we overexpressed WFDC2 gene in proximal tubular cell line (HK2) and collected its culture media (Figure 5E). Then, we used the culture media that contained secreted WFDC2 proteins to treat podocyte cells and GECs. We found that PEX19's mRNA and protein levels both decreased in podocyte cells and GECs with WFDC2 treatment (Figure 5F).

## Discussion

Integrative informatics approach is a powerful tool to explore the pathogenesis and to identify the therapeutic targets for complex diseases (45). The informatics approaches that combine high-throughput data with the identification of DEGs interacting networks and pathways could drive kidney diseases. Advances in omics biotechnology, such as next-generation DNA sequencing and protein mass spectrometry, let us study the complex CKD in genome, transcriptome, and proteome levels to identify the interaction between molecules that play synergistic roles (45). Here, in this study, we used integrative informatics analysis that identified the DEGs interactions from the tubules and glomeruli that play pathological roles in CKD processes. This pattern of study we used with the combination of experimental approaches and informatics approach is expected to provide us with a deeper understanding of the interaction of critical genes to elucidate CKD progression and could be new potential therapeutic targets.

The physiology of kidney function and the pathophysiology of kidney disease involve interactions of different cells from the tubules and glomeruli of the kidney. Many studies have shown that tubular injury can cause subsequent glomerular injury. Tubulointerstitial hypoxia caused by peritubular capillary loss stimulates fibrogenesis with increased collagen I and a-smooth muscle actin, indicators of increased myofibroblasts (46, 47). HIF-2a target genes are upregulated in sclerosing glomeruli, and there is a potential signaling interaction between transforming growth factor beta and hypoxia-inducible factors (HIFs) to promote renal fibrogenesis, even in normoxia (48). Meanwhile, other studies have delineated numerous mechanisms whereby glomerular injury causes tubulointerstitial injury, including ischemia, filtered proteins/cytokine elaboration, and so on (49). The tubular injury and glomerular injury feedbacks enhance CKD progression in all settings, whether there is initial isolated tubulointerstitial injury or combined glomerular/tubular injury. However, the initial or consequence of tubular or glomerular injury to the other component is not well-studied. The interaction of genes or proteins in the tubules and glomeruli is still less known. In this study, we performed correlation analysis to identify the associated genes in the tubules and glomeruli and verified their interaction *via* experiment, providing a methodology pipeline of the identification of interaction genes in the tubules and glomeruli.

DKD remains as the most common cause of end-stage renal disease (ESRD) in the US and most countries (50). DKD is most likely a disease with individual and temporal heterogeneity. Pathological and molecular understanding of this heterogeneity will be essential to make progress (50). HE4 (encoding human epididymis protein 4, also known as WAP 4-disulfide core domain-2 or WFDC2) is a secretory protein produced in normal glandular epithelium of the reproductive tract, renal tubules, and respiratory epithelium (51). A study showed that WFDC2 circulating WFDC2 is postulated to be a biomarker of renal fibrosis in DKD patients (52). Another study also showed that serum WFDC2 is associated with renal function and DKD in patients with type 2 diabetes mellitus (53). The overexpression of HE4 in serum from CKD patients was associated with decreased kidney function, and the serum concentrations of HE4 obviously increased with advanced renal fibrosis stage in patients with CKD (54). Increased HE4 in serum is closely associated with the development of LN or CKD in patients with systemic lupus erythematosus (55). Recently, a study identified HE4 as a fibroblast-derived mediator of fibrosis, as an inhibitor of multiple proteases, including serine proteases and matrix metalloproteinases, and as a specific inhibitor of their capacity to degrade type I collagen (43). Another study indicated that HE4 in TECs promotes extracellular matrix accumulation and renal fibrosis *via* nuclear factor kappa B (NF-kB) (31909536). WFDC2 was also reported to play important roles in diabetes and DKD. Our study found that activated WFDC2 in the tubules could interact with glomerular podocyte/endothelial cells, causing the downregulation of peroxisomal biogenesis factor 19 PEX19 mRNA and protein levels and effect downstream pathways. PEX19 undoubtedly is a key player in several steps of peroxisomal membrane proteins (PMPs) transport (56). Here, in this study, our data showed that WFDC2 was upregulated in the tubules causing PEX19 expression to decrease in the glomeruli, which provide a new mechanism of how WFDC2 regulates diabetes and DKD.

There are many other known genes associated between the tubules and the glomeruli that enhanced kidney diseases from identification. We identified that the mRNA level of C1QB in the tubules is correlated with the mRNA level of IRF8 in CKD patients. Other studies have shown that C1qB promoter was co-precipitated with PU.1 and IRF8. shRNA knockdown of PU.1 and IRF8 diminished C1qB promoter response to interferon gamma (IFNγ). STAT1 instead regulated C1qB promoter through IRF8 induction (32). We also identified that TIMP1 and TGFBI are associated in the tubules and glomeruli, and that the association is confirmed by other studies (57). However, our analysis showed that these two genes are positively correlated in the tubules and glomeruli in CKD. Meanwhile, the TGFBI overexpression in human corneal epithelial cells result in MMP1, MMP3 increasing, and TIMP1 decreasing. More experimental study needs to be performed to study their relationship.

Our study has limitations. Firstly, as there are more single-cell sequencing experiments performed by many groups, it is very direct to analyze the transcriptome of different cell types and identify the association genes. However, there are still issues that needed to be improved for single-cell sequencing technology, such as low depth of the sequencing and artificial bias caused by process steps for obtaining single cells. Therefore, the method of bulk sequencing data analysis for the tubules and glomeruli can obtain some information that single-cell sequencing cannot identify. Secondly, we used all kinds of CKD patients for analysis, including LN, DKD, FSGS, MN, IgAN, and MCD. We realize that there should be a huge variation between the mechanisms of different disease types. However, with more sample number and robust integrative informatics approach, we can identify the common critical genes or mechanistic pathways in all kinds of CKD progression.

## Data Availability Statement

The datasets generated for this study can be found in online repositories. The names of the repository/repositories and accession number(s) can be found in the article/Supplementary Materials.

## Ethics Statement

The animal study was reviewed and approved by the First Hospital of Jilin University Ethics Committee.

## Author Contributions

JH and CW led the project, designed the study, analyzed and interpreted the data, and drafted the manuscript. ZY, LL, and FM performed the meta-analysis and bioinformatics analysis. LL, FM, YH, XY, and BX performed the experiments of qPCR, western blot, cell culture, and mice model. JH and FM performed other statistical analysis. JH and CW performed the study conception and design, along with drafting of the manuscript. All authors contributed to the article and approved the submitted version.

## Conflict of Interest

The authors declare that the research was conducted in the absence of any commercial or financial relationships that could be construed as a potential conflict of interest.
