# Supplementary material for: Integrative Informatics Analysis of Transcriptome and Identification of Interacted Genes in the Glomeruli and Tubules in CKD
Source: Front Med (Lausanne). 2021 Feb 12;7:615306. doi: 10.3389/fmed.2020.615306 (PMC7906987; doi:10.3389/fmed.2020.615306)
Supplement: Supplementary file 2 [file Presentation_1.pptx]

## Slide 1
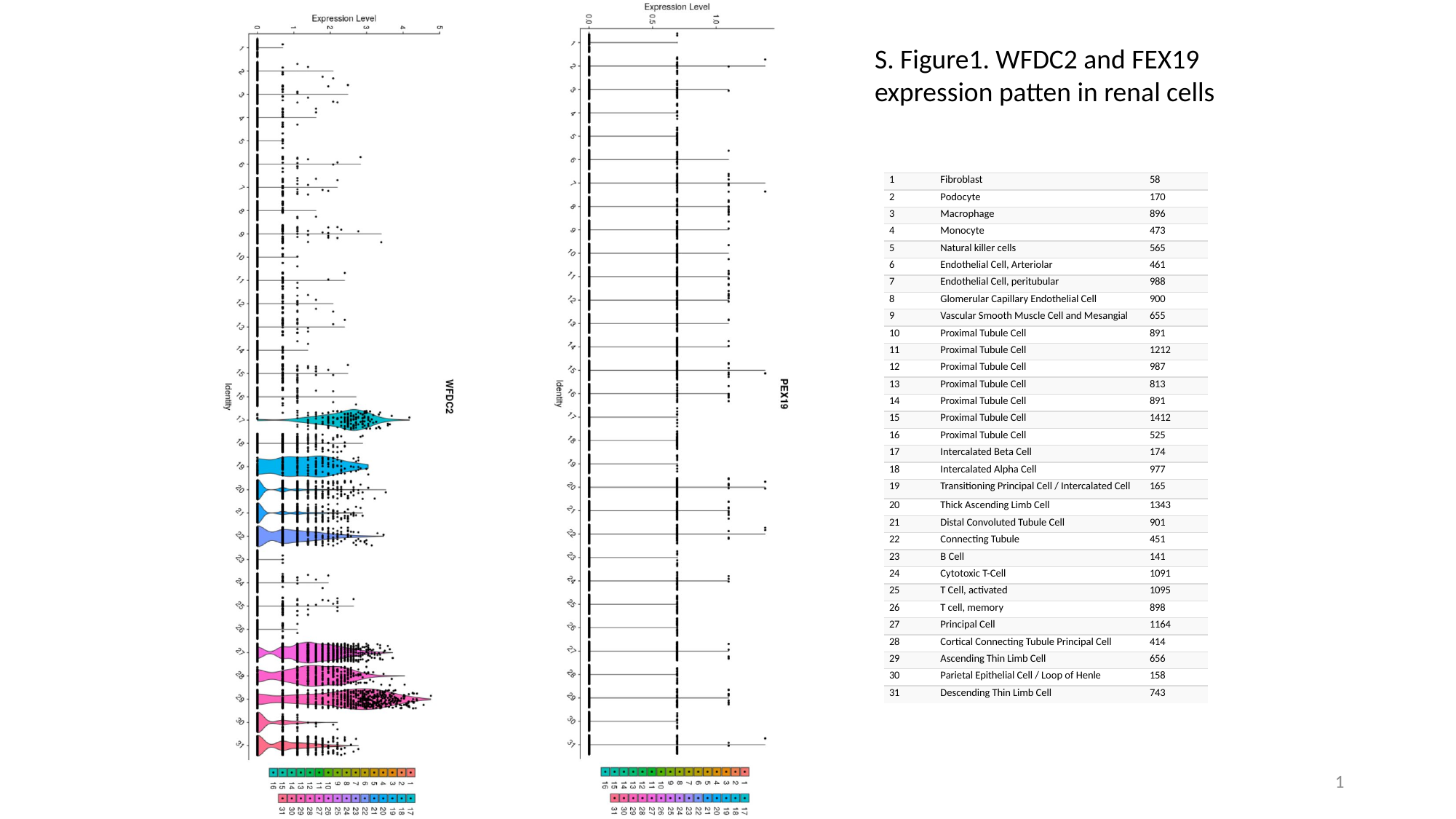

S. Figure1. WFDC2 and FEX19 expression patten in renal cells
| 1 | Fibroblast | 58 |
| --- | --- | --- |
| 2 | Podocyte | 170 |
| 3 | Macrophage | 896 |
| 4 | Monocyte | 473 |
| 5 | Natural killer cells | 565 |
| 6 | Endothelial Cell, Arteriolar | 461 |
| 7 | Endothelial Cell, peritubular | 988 |
| 8 | Glomerular Capillary Endothelial Cell | 900 |
| 9 | Vascular Smooth Muscle Cell and Mesangial | 655 |
| 10 | Proximal Tubule Cell | 891 |
| 11 | Proximal Tubule Cell | 1212 |
| 12 | Proximal Tubule Cell | 987 |
| 13 | Proximal Tubule Cell | 813 |
| 14 | Proximal Tubule Cell | 891 |
| 15 | Proximal Tubule Cell | 1412 |
| 16 | Proximal Tubule Cell | 525 |
| 17 | Intercalated Beta Cell | 174 |
| 18 | Intercalated Alpha Cell | 977 |
| 19 | Transitioning Principal Cell / Intercalated Cell | 165 |
| 20 | Thick Ascending Limb Cell | 1343 |
| 21 | Distal Convoluted Tubule Cell | 901 |
| 22 | Connecting Tubule | 451 |
| 23 | B Cell | 141 |
| 24 | Cytotoxic T-Cell | 1091 |
| 25 | T Cell, activated | 1095 |
| 26 | T cell, memory | 898 |
| 27 | Principal Cell | 1164 |
| 28 | Cortical Connecting Tubule Principal Cell | 414 |
| 29 | Ascending Thin Limb Cell | 656 |
| 30 | Parietal Epithelial Cell / Loop of Henle | 158 |
| 31 | Descending Thin Limb Cell | 743 |
1
